# Web-Based Interventions Alone or Supplemented with Peer-Led Support or Professional Email Counseling for Weight Loss and Weight Maintenance in Women from Rural Communities: Results of a Clinical Trial

**DOI:** 10.1155/2017/1602627

**Published:** 2017-04-05

**Authors:** Patricia A. Hageman, Carol H. Pullen, Melody Hertzog, Bunny Pozehl, Christine Eisenhauer, Linda S. Boeckner

**Affiliations:** ^1^Physical Therapy Education, College of Allied Health Professions, University of Nebraska Medical Center, 984420 Nebraska Medical Center, Omaha, NE 68198-4420, USA; ^2^College of Nursing, University of Nebraska Medical Center, 985330 Nebraska Medical Center, Omaha, NE 68198-5330, USA; ^3^College of Nursing, University of Nebraska Medical Center, Lincoln Division, 1230 “O” Street, Suite 131, Lincoln, NE 68588-0220, USA; ^4^College of Nursing, University of Nebraska Medical Center, Northern Division, 801 E. Benjamin Avenue, Norfolk, NE 68701-0469, USA; ^5^Nutrition and Health Sciences, College of Education and Health Sciences, University of Nebraska-Lincoln, 110 Leverton, Lincoln, NE 68583-0806, USA

## Abstract

*Objective*. This trial compared the effectiveness of a web-based only (WO) intervention with web-based supplemented by peer-led discussion (WD) or professional email counseling (WE) across 3 phases to achieve weight loss and weight maintenance in women from underserved rural communities.* Methods*. 301 women (BMI of 28–45 kg/m^2^) randomly assigned to groups participated in guided weight loss (baseline to 6 months), guided weight loss and maintenance (6 to 18 months), and self-managed weight maintenance (18 to 30 months).* Results*. Retention was 88.7%, 76.5%, and 71.8% at 6, 18, and 30 months, respectively. Intent-to-treat analyses demonstrated no group differences in change in weight within any phases. At 6 months, observed mean (SD) weight loss was 5.1 (6.0) kg in WO, 4.1 (5.6) kg in WD, and 6.0 (6.3) kg in WE, with 42%, 38%, and 51%, respectively, meeting ≥ 5% weight loss. These proportions dropped by a third after phase 2 with no further change during phase 3.* Conclusion*. Web-based interventions assisted women from rural communities in achieving 6-month weight loss, with weight regain by half at 30 months. No group differences were potentially due to the robust nature of the web-based intervention.* Trial Registration*. This trial is registered with ClinicalTrials.gov NCT01307644.

## 1. Introduction

Women from rural communities represent one of the largest medically underserved populations in the United States, with high rates of overweight and obesity, physical inactivity, and poor diets placing these women at high risk for developing chronic diseases, such as diabetes, heart disease, and stroke [1–3]. Behavioral approaches for lifestyle modification are recommended for treating overweight and obesity, yet women from rural communities have limited access to preventive services, such as counseling in diet and activity [3–5]. The 2011 Strategic Plan for the National Institutes of Health Obesity Research highlighted the need for designing and testing innovative lifestyle interventions to reach underserved rural populations, who face unique barriers to healthy behaviors [6].

The number of web-based lifestyle behavior change interventions targeting weight loss and weight maintenance has dramatically increased over the past 10 years, in part due to increased web accessibility and its potential for being a cost-effective method to address behavior change in hard-to-reach populations [7–9]. The majority of studies of both weight loss and weight maintenance focus on web-based delivery as a supplement to face-to-face counseling or as an alternative to face-to-face counseling [7, 8]. Web-based interventions result in smaller weight losses and lower levels of weight maintenance compared to in-person interventions, though small weight changes may be clinically relevant on a public health level as compared to no interventions [7, 8]. Few studies have investigated interventions delivered solely via web, or web-based with additive elements, and/or of a duration greater than four to six months [7, 8].

The challenge with any lifestyle modification intervention is to engage the participants to achieve 5% to 10% of body weight loss, the percentage loss associated with health benefits [10, 11]. Keeping participants engaged over longer periods of time, as needed for weight loss maintenance, presents greater issues and challenges, as weight regain occurs consistently within six to nine months of initial weight loss for most individuals [12, 13]. Maintaining engagement in purely web-based weight loss and weight maintenance interventions may be particularly challenging, though reviews of technology-based studies identified strategies that may be effective such as self-monitoring, goal setting, theory-based behavior change messaging, social support, and skill building to prevent weight regain [13–16]. In multiple studies of web-based weight loss and weight maintenance, greater adherence or intervention dosage, as measured by attendance in online chats or login frequency, was associated with weight change [17–20]. In face-to-face lifestyle weight loss interventions, additional support from peers or professionals enhanced participant engagement, adherence, and weight outcomes [13, 16, 21]. Providing additional support such as email counseling and peer-led discussion in web-based studies could potentially boost participant engagement and improve weight loss outcomes [9, 16, 21, 22].

Web-based weight loss and/or weight maintenance studies that augmented a web-based only group with individualized feedback in the form of email counseling, e-coaching, or automated computer feedback had varying results, possibly due to differing intervention durations (three to 12 months), though participants receiving individual feedback at three months were observed to lose more weight than those receiving no additional feedback, and the effect was slightly greater for email counseling [7, 17–20, 23]. Outcomes of other web-based studies using supplemental support via professional email counseling showed promising yet inconclusive results [24–26].

Studies of the effectiveness of web-based peer-led support groups varied in how the support was provided, as some provided peer support by health professionals, and others used multicomponent interventions where the web-based peer component effects were not separated for analysis [8, 18, 20, 24]. In our three-month pilot study of women aged 50–69 from rural communities, we found that access to a web-based intervention supplemented with a peer-led asynchronous support group doubled the engagement with the web intervention and enhanced weight loss compared to a web-based only group [27].

Few studies have compared purely web-based weight loss and weight maintenance interventions to those with enhanced features or have targeted women from rural communities, considered a designated priority population for study due to documented health disparities [28]. This study is unique, as all three web-based intervention groups receive three specific intervention phases progressing from intensive guided weight loss tapering to guided weight maintenance and finally self-management of weight maintenance, and the study offers longer weight maintenance interventions, as recommended by the Institute of Medicine for optimal long-term outcomes [29, 30].

The conceptual framework of this study was based upon Pender's Health Promotion Model (HPM), which is founded on social cognitive theory, in order to explain and modify the adoption and maintenance of healthy eating and activity behaviors necessary to achieve weight loss and weight maintenance [31]. The intervention messaging for behavior change focused on perceived benefits of action, perceived barriers to action, self-efficacy, and interpersonal influences of family and friends for action, all of which are behavior-specific cognitions from the HPM. The interventions also focused on the commitment to a plan of action through goal setting.

The primary aim of the study was to compare the effectiveness of a web-based only (WO) intervention with web-based interventions supplemented with peer-led discussion support group (WD) or professional email counseling (WE) for achieving change in primary outcomes of body weight and waist circumference and secondary outcomes of attaining criterion weight loss targets (5% and 10% weight loss) by phase: phase 1 for guided weight loss (baseline to 6 months), phase 2 for guided continuing weight loss and maintenance (6 to 18 months), and phase 3 for self-managed weight maintenance (18 to 30 months).

The secondary aim was to compare the three groups on improvement in primary outcomes of healthy eating and activity (Kcal intake, weekly minutes of moderate or greater intensity activity, blood pressure, and lipids), as well as achieving secondary outcomes of eating and activity at recommended criterion targets (20–35% of daily fat kcal intake and 150 weekly minutes or greater of moderate or greater physical activity) by the end of each of the three phases.

Based upon the literature and our pilot work, we hypothesized that each of the intervention groups receiving supplemental elements, either WD or WE, would have better outcomes than the WO group. The evidence in the literature was insufficient to hypothesize the relative effectiveness of the peer-led discussion support group compared to the professional email counseling.

## 2. Methods

Ethical approval for this study was received through the Institutional Review Board of the University of Nebraska Medical Center (approval number: 23710-FB). Written informed consent was obtained from all participants, following the standardized protocol prior to their enrollment. The study was conducted between June 2011 and December 2014, with recruitment, enrollment, and baseline assessments occurring over one year (June 2011 to May 2012). Eligible women were randomly allocated to 1 of 3 parallel intervention groups (1 : 1 : 1 ratio), with each group receiving three intervention phases over a 30-month period [29].

### 2.1. Participants

A total of 301 women, aged 40–69, who met rural status as defined by the Rural-Urban Commuting Area (RUCA) codes [32], which classifies rural based upon population density and population work commuting patterns, were recruited from northeastern Nebraska in the USA. The study specifically targeted women, as opposed to both genders, in response to longitudinal data showing women having a disproportionately higher rate of late-life disability than men, which might be reduced through gender-appropriate preventive interventions earlier in life [33].

Women were eligible for the study if they had a BMI of 28–45 kg/m^2^ and were not taking medications that affected weight loss or weight gain, as verified by research personnel. Other inclusion criteria included ability to speak and read English, communicate over the phone, and use a computer including email features with minimal assistance. They also needed to have access to the Internet and a DVD player and be willing to drive up to 70 miles each way to our centrally located research office at a community college for all required assessments. Eligible women were included if they answered “no” to all questions on the Physical Activity Readiness Questionnaire (PAR-Q) or obtained medical clearance from their physician to participate [34]. Major exclusion criteria were as follows: having a diagnosis of type 1 diabetes or having a diagnosis of type 2 diabetes and requiring insulin, having 10% or greater weight loss in the last six months, being currently enrolled in a weight loss management program or another research study, participating in current cancer treatment, or having physical or medical restrictions that would preclude following recommendations for moderate activity and healthy eating.

### 2.2. Trial Conduct

A project recruiter was hired from the region to assist in recruiting women through local advertising and direct mailings, with special efforts to recruit persons from underrepresented racial/ethnic backgrounds and/or individuals with lower socioeconomic status through local community contacts. Women expressing interest in the study received a phone follow-up screening interview conducted by the recruiter or other study personnel to verify that all eligibility requirements were met.

Research nurses completed the informed consent process with eligible women at the assessment site. Baseline assessments were conducted over two visits scheduled within three weeks. At baseline visit one, nurses provided each woman with a pedometer [Omron HJ-112 GoSmart, Omron Healthcare, Inc., 1925 W Field Ct, Lake Forest, IL 60045] and an accelerometer, with instructions for their use. Nurses dispensed book of food counts [35] and a web user's guide to women (designed to assist women in accessing and using the intervention website). At completion of the first baseline visit, nurses delivered a sealed confidential envelope to each woman that contained an identification number and a password for logging into the intervention website and advised women to keep their login information materials confidential. Women were informed of a “practice period” between baseline visits one and two during which they would be asked to access and become acquainted with their intervention website by logging weight, food intake, and pedometer steps. The intent of the practice period was to further inform the women about the nature of the project, assess their willingness to self-monitor, and verify computer and web literacy. If the women did not participate in the practice period as noted by the technical support team, they were dropped from the study. At the second baseline visit, women repeated selected measures for reliability purposes (e.g., blood pressure) and performed any remaining assessments not completed during baseline visit one.

#### 2.2.1. Randomization and Blinding

A randomization schedule was created by the project statistician using online software to generate sequences of pseudorandom numbers (http://www.randomizer.org/form.htm). To keep accrual relatively even during rolling enrollment, a random ordering of block sizes 12, 15, and 18 was used. The project statistician did not have any contact with the women during the trial. Upon completion of baseline assessment visit two, each woman received an electronic notice on her intervention web account of her group assignment. The women were instructed to not share this with others, including the research nurses who conducted the assessments, who were blinded to web-intervention content as well.

## 3. Interventions

### 3.1. Web Intervention for All Groups

Women in all three groups had access to the same content on the intervention website, except that two groups received either a supplemental peer-led discussion blog or professional email counseling. All women received a behavior change lifestyle plan on the website, consisting of eating and activity recommendations based upon the 2010 Dietary Guidelines for Americans [36], the 2008 Physical Activity Guidelines for Americans [37], and Healthy People 2010 [38]. Dietary recommendations included reduction in caloric intake (range 1,200 to 1,600 Kcal), an average daily fat intake of 20–35%, and a focus on nutrient-dense food and beverages [36, 38]. For physical activity, women were encouraged to achieve a criterion activity level of ≥150 minutes weekly of moderate or greater activity, and if this was achieved, they were encouraged to increase their activity to 60 to 90 daily minutes of moderate or greater physical activity as recommended for weight loss maintenance [39, 40].

Table 1 summarizes the interventions delivered to each of the three groups. Intervention details are included in the protocol paper [29], and specific web content denoted by theoretical construct for each time point of delivery by phase is reported on the clinical trial website (http://unmc.edu/alliedhealth/research/ww4w). Sample website screen views are included in additional files [see Supplemental Illustration 1 in Supplementary Material available online at https://doi.org/10.1155/2017/1602627]. Women had access to technology support throughout the entire 30-month study from the project technologist.

### 3.2. Peer-Led Discussion Group

Women in the WD group could view an extra tab on the intervention home page labeled “Discussion.” This asynchronous discussion board was managed by a peer-leader, trained by the project investigators, who was responsible for posting new theme-based messages, called primers, on a predetermined schedule by phase, as noted in Table 1. When posting message responses, each participant had a unique and nonpersonalized identifier. A detailed listing of the content of discussion board posts by phase is located on the trial website (http://unmc.edu/alliedhealth/research/ww4w).

### 3.3. Professional Email Counseling

Women in the WE group received emails from a registered dietitian whose identity was masked. The email counselor was responsible for reviewing the WE women's web-based logs of weight, eating, activity, and goal setting and sending an email with feedback using a structured process [41] using a frequency which was consistent with the WD group “primers” during phases 1 and 2 (see Table 1). The WE participants were allowed, but not required, to respond to any email they received from the professional counselor. During phase 3, the women were notified that they could email the professional counselor at any time with questions; however, the email counselor only responded to participant-initiated emails.

### 3.4. Intervention Unexpected Events

The only major unexpected event that may have influenced intervention delivery was a technical issue that occurred on April 21, 2012, nearing the May 2012 completion date for enrollment, when the server which housed the study's website upgraded its security, making computers using one of the default settings in Internet Explorer (TLS 1.0) unable to read the website. The problem was discovered on April 22, and the technical support team made contact with women to rectify the issue with verification that all women were able to log in by May 11, 2012.

### 3.5. Outcomes

Table 2 outlines the primary and secondary outcomes for the primary aim (weight) and secondary aim (eating and activity), with a listing of the measurement instruments used and frequency of the assessments. The details of the assessment and measurements have been published previously [29], and a brief summary is provided below. Women completed surveys of general demographic information and health history. Measures of height and weight were assessed using the Tanita scale [TBF-215, Tanita Corporation of American, Inc., 2625 S Clearbrook Dr., Arlington Heights, IL 6005-9824]. BMI was calculated as weight in kilograms divided by height in meters squared. Waist circumference was measured using a snug tape parallel to the floor but held without skin compression around the abdomen located at the level of the iliac crest at the end of expiration, with the average of two trials recorded [42].

The key behavioral measures related to eating (i.e., kcal intake daily, percent daily calories from fat) were assessed using the web version of the 1998 Block Health Habit and History Questionnaire, which asked for the frequency of consumption of particular food items during the last three months [43]. Prior research has shown this instrument to demonstrate high reliability, validity, and sensitivity to change [44–46].

Standardized methods were followed to assess women's estimated daily minutes of moderate or greater physical activity over seven days using the lightweight triaxial Actigraph Accelerometer (Model GT3X, Pensacola, FL), shown to have established reliability and validity [47–49]. Women were asked to wear the Actigraph on the dominant hip attached to an elastic waist band for 24 hours a day, except when showering or swimming, over seven days. Adherence to the wear guidelines was high at all times. Mean days of wear ranged from 6.8 (SD = 0.6) at baseline to 6.6 days (SD = 1.2) at 30 months, with the percentage of women wearing the Actigraph on six or seven days exceeding 94% at all times.

Self-reported weekly minutes of moderate or greater physical activity were assessed using the Behavioral Risk Factor Surveillance System (BRFSS) Physical Activity module consisting of seven items that have been shown to have acceptable reliability and validity [50]. Following the methods used by the Women's Injury Study, the BRFSS questions were administered to our women to estimate the weekly minutes of moderate or greater physical activity [51].

Biomarkers affected by eating and activity included blood pressure and blood lipids. Following 5 minutes of quiet sitting, blood pressure was measured using standardized methods [52]. Women were asked to fast for 12 hours prior to assessments to determine fasting blood lipids that required a blood draw by the research nurses and were processed using a standardized protocol [53].

Other data, including women's perceptions of behavioral determinants and process evaluations via surveys and focus groups, were collected as described previously [29]. These results will be reported in a future paper.

### 3.6. Website Usage

Tracking of the women's access to and use of the intervention website occurred by documenting the number of logins, including date and time, and their “clicks” on various features within the website, including clicks on the discussion board items for those enrolled in the WD group. The proxy for dosage was the count of women's logins by time points of new content, regardless of whether they accessed one or more of the web features or whether they logged in one or multiple times during a given week. For example, a woman who logged in on each of the 26 weeks of new web content during phase 1 was considered to have 100% dosage. Dosage of use of the discussion board was defined similarly, noting women's clicks specific to the discussion board site by time points of new primers posted by the peer-leader. For those in the WE group, monitoring of the number of emails sent by the professional email counselor and the replies from participants was logged; however, the planned protocol to use the email return receipt feature to monitor women's opening of email messages was found to be not feasible.

### 3.7. Sample Size

As detailed previously, the sample size of 306 was based on the most conservative planned test of group difference in average change in primary outcomes from beginning to end of a phase using generalized estimating equations [29]. This would provide power of at least .80 using *α* = .017 for a two-tailed test of a mean difference of .45 standard deviations, the median effect size for weight and waist circumference found for this comparison in prior studies. Based on our pilot work, an effect of this magnitude was also plausible for group differences in change in physical activity outcomes, healthy eating behaviors, blood pressure, and lipids. The sample size also allowed for an estimated 25% attrition over the course of the study. The revised analysis using linear mixed model methods to estimate a mixed-effects repeated-measures model maintained power of at least .80 with our observed data for the same effect size and planned comparisons.

In our National Institutes of Health study proposal, we followed an accepted strategy of estimating a priori power only for the analysis of primary outcomes, acknowledging that analyses of secondary outcomes were likely to have less power but would be conducted for their descriptive value. However, the power of a two-tailed *z*-test of pairwise differences in estimated proportions with *n* = 100 per group and *α* = .017 is .80 if the difference is approximately .22. The GEE analysis with imputed data, which also took into account dependence of observations across times, might be expected to have power of .80 for a somewhat smaller difference.

### 3.8. Data Analysis

The SPSS v.23 Missing Values Analysis module was used to evaluate missing data. Nearly all cases had complete data at baseline, with two women (0.7%) missing actigraphy variables and 1 (0.3%) missing lipids. Statistical comparisons of women with complete data (*n* = 208) and those with at least one missing data point (*n* = 93) found the groups similar in baseline marital status, education, Internet access, comfort with using computers, waist circumference, and activity. However, women with missing data were approximately 2.4 years younger and 4.5 kg heavier and consumed 170 more calories per day at baseline. Weight loss for dropouts was not significantly different for completers as compared to noncompleters at 18 months (.4 kg less, *p* = 0.879).

Each intervention phase had distinct theoretically derived elements, and the primary analysis tested pairwise comparisons of the intervention groups on average change within each phase: (a) WO with WD using a one-sided test, (b) WO with WE using a one-sided test, and WD with WE using a two-sided test. Because these comparisons were nonorthogonal, each was tested at *α* = .017 (.05/3).

A maximum likelihood approach was used for the analysis of the primary (continuous) outcomes. With fixed and common measurement times (time treated as categorical), a mixed-effects repeated-measures model can be fit using linear mixed model methods, specifying a random intercept and unstructured variance/covariance error matrix [54]. Partial cases are included in this analysis under the assumption that missing observations are missing at random (MAR). Each participant's data were analyzed according to her randomized assignment, regardless of adherence to protocol. Data from all cases and at all times (including intermediate assessments, if available) were used to estimate the model, which incorporated contrasts to test pairwise group differences from the beginning to the end of each phase. Supplemental tests of simple effects, conducted using a *p* of 0.05, were used to evaluate change within each group.

Secondary outcomes for aims 1 and 2 were attainment of criterion weight loss and eating and activity targets, dichotomous outcomes having nonnormal distributions. Thus, generalized estimating equations were used to perform the analysis. For the subset of outcomes needed in order to determine whether criteria were met, multiple imputation of missing data was carried out using SPSS Missing Values Analysis under the assumption that data were missing at random. Imputation was performed separately for each intervention group in order to preserve any interactions of group with the other variables. Two baseline variables were included as auxiliary variables: age, which had modest correlations with many variables, and perceived barriers to healthy eating, which was significantly associated with missingness. No variables were transformed prior to imputation [55]. A fully conditional specification (FCS) method was used for all variables, and 20 imputed datasets were created. After imputation, dichotomous variables indicating whether or not criteria were met were calculated. Using SAS 9.3, generalized estimating equations with a binomial error distribution and logit link were used to test pairwise group differences in the proportion of women meeting these goals at the end of each phase (6, 18, and 30 months), accounting for the nonindependence across time. As with the linear mixed model, time was treated as categorical. PROC MIANALYZE in SAS was used to combine estimates across imputed datasets.

Intervention dosage was analyzed by phase with descriptive statistics, tabulating the frequency and percentage of women completers in each group by operationally defined dosage levels based on the number of weeks in which they logged on to the website. In addition, the correlation of number of logins and weight loss by 30 months was calculated. For the WD group, dosage of posting to the discussion board was also calculated.

## 4. Results

### 4.1. Participant Flow

**Figure 1:**
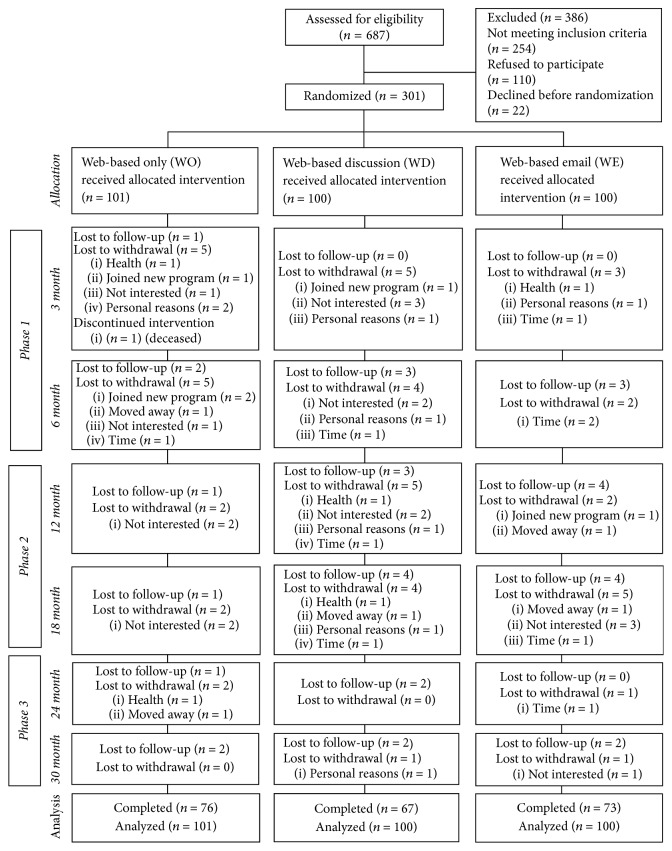
Participant flow diagram of the Women Weigh-in for Wellness clinical trial.

Of 687 women assessed for trial eligibility, 323 women were enrolled and 301 women randomized after participating in the practice period and completing the baseline two visit (see Figure 1). Retention rates were 88.7%, 76.5%, and 71.8% between baseline and each of 6 months, 18 months, and 30 months, respectively, with no significant differences in retention rates across groups. Of those who dropped from the study, the primary reasons for withdrawal were lost to follow-up (*n* = 28), not interested/did not wish to continue (*n* = 11), and time commitment (*n* = 9).

### 4.2. Baseline Characteristics

The women were randomized into WO (*n* = 101), WD (*n* = 100), and WE (*n* = 100) groups. Baseline characteristics did not differ among the groups (see Table 3). Collectively, the mean age (SD) was 53.9 (6.9) years. Based upon BMI, 32 (10.6%) were overweight, 143 (47.5%) were obese I, 85 (28.2%) were obese II, and 41 (13.6%) were obese III. Baseline characteristics showed that the majority were Caucasian (97.3%; *n* = 293), were employed full-time (68.8%; *n* = 207), and had a college degree or higher education (84.4%; *n* = 254). The majority (77.1%; *n* = 232) reported having a household income > $40,000. Overall, the women were healthy, with 106 (35.2%) reporting having no comorbidities, 95 (31.6%) reporting one comorbidity, and the remainder having two or more comorbidities. The most common comorbidities were the general health issues of anxiety, depression, or migraines (34%, *n* = 105) and arthritis (26.6%, *n* = 80).

### 4.3. Aim 1: Weight

#### 4.3.1. Primary Outcomes

The intervention groups did not differ significantly from each other in mean change on either of the primary outcomes (body weight and waist circumference). This finding was consistent across all phases and for all pairwise comparisons (see Tables 4 and 5). Considering simple effects within group, all means on both primary outcomes decreased significantly from baseline to 6 months. Across the groups, estimated mean weight loss at 6 months ranged from 4.0 to 5.8 kg, representing from 4.2% to 6.2% of initial body weight. Mean decreases in waist circumference ranged from 4.6 to 6.2 cm. However, from 6 months to 18 months, there was significant increase in all groups, with average weight increasing by approximately 2 kg or from one-third to nearly one-half of phase 1 loss. On average, there was further significant weight gain of about 1 kg from 18 months to 30 months. Waist circumference also significantly increased during phase 2.

#### 4.3.2. Secondary Outcomes

The estimated percentage of women in each group who lost at least 5% of their baseline weight by the end of phase 1 was 42% in the WO group, 38% in the WD group, and 51% in the WE group. Percentages for meeting the criterion of at least 10% weight loss were 24%, 19%, and 29%, respectively. None of the pairwise comparisons were significant for these outcomes in any of the three phases. The proportion meeting each of these criteria dropped by about one-third from the end of phase 1 to the end of phase 2, but almost no further change occurred during phase 3 (see Table 6).

### 4.4. Aim 2: Eating and Activity

#### 4.4.1. Primary Outcomes

Within each phase, there were no significant pairwise comparisons for any of the primary outcomes (Kcal intake, weekly minutes of moderate or greater intensity activity, blood pressure, or lipids), with the exception of a larger improvement in HDL seen in the WO group compared to the WE group in phase 1. Effects favoring the WO group had not been hypothesized (see Table 5).

Simple main effects tests for eating and activity measures showed the desired change that occurred in self-reported activity, kcal intake, triglycerides, and HDL during phase 1. Systolic and diastolic blood pressure showed significant decreases in phase 1 in the WE group, but only systolic blood pressure decreased in the WO group. Both groups maintained these improvements in phases 2 and 3.

#### 4.4.2. Secondary Outcomes

A significantly higher proportion of the WE group than the WD group met the standards for % of calories from fat at 6 months (estimated proportions of .56 versus .38). No differences were observed between groups in the proportion of individuals meeting the physical activity targets, whether measured by Actigraph or self-report, in any of the phases.

### 4.5. Website Usage and Weight Change

Logins by week of new web-intervention content were used to estimate the dosage of web intervention received (Table 7). The pattern of participation in the basic website was similar among women in all groups for phase 1, with 69.0%, 65.9%, and 69.6% of 6-month completers in WO, WD, and WE groups, respectively, logging in for ≥50% dosage. The dosage of web intervention received declined by approximately half during phase 2 and two-thirds in phase 3. Weight change was significantly related to the website utilization (*r* = −0.25, *p* < 0.01), with greater participation correlated with greater success with weight loss and weight maintenance over the trial.

Among women in the WD group, nearly half (45%) viewed and/or posted on the discussion board for a dosage of 50% or more when new discussion board primers were made available during phase 1. Women's use of the discussion board dropped dramatically during phase 2, with only about 22% having a dosage of 50% or more. During phase 3, the majority of women (58.2%) in the WD group never viewed the discussion board.

For the WE group, the intervention delivery was 99.9%, meaning that the targeted number of messages was sent to the participants at the scheduled time. Eighty-nine percent and 69% of WE participants sent email responses to the email counselor during phases 1 and 2, respectively. During phase 3, the WE group no longer received emails from the counselor, but 16% of these women sent emails to the counselor during that 1-year self-management phase.

## 5. Discussion

The Women Weigh-in for Wellness community-based clinical trial compared the effectiveness of a web-based only intervention (WO) with two web-based interventions with additional elements (WD and WE) on achieving initial weight loss (baseline to 6 months), guided weight loss and weight maintenance (6 to 18 months), and self-management of weight maintenance (18 to 30 months), with findings that, on average, women from rural communities in all groups achieved the majority of weight loss at 6 months (representing 4.2% to 6.2% of initial weight loss), with gradual regain of approximately half the weight lost by 30 months. There were no differences in this outcome among the three web-based groups (WO, WD, and WE) within any of the phases. The 6-month estimated group mean weight losses ranged between 4.0 and 5.8 kilograms, with 42% (WO), 38% (WD), and 51% (WE) of women meeting the 6-month target of ≥5% of initial body weight loss. All three groups showed a drop of approximately 10 percentage points in the number of women meeting the ≥5% loss criterion by 30 months.

The observed pattern of our women achieving the most rapid weight loss during phase 1 guided weight loss (baseline to 6 months), with women showing a slow rate of regain during phase 2 (6 to 18 months) and phase 3 (18 to 30 months), is consistent with findings of a systematic review and meta-analysis of weight loss clinical trials with a minimum one-year follow-up conducted by Franz et al. [56]. As noted in a Cochrane Systematic Review of web-based weight loss and weight maintenance interventions [7], the different rate of weight change suggests that weight loss and weight loss maintenance interventions need to be considered separately, as was done in this study.

The amount of weight change among women in this study, particularly at the 6-month endpoint, is consistent with the ranges reported among other purely web-based studies [17, 20, 26]. Overall, an estimated 43.7%, 34.7%, and 34.3% of women at 6, 18, and 30 months, respectively, met the weight loss target of ≥5%, the amount recommended for health benefits. Although weight loss was modest, there is evidence that suggests that some degree of weight loss, even if it is not sustained beyond 6 months, may prove beneficial for blood pressure [11]. Weight losses below 5% may be of clinical significance on a public level, as for each kg of weight loss, a meta-analysis of 25 studies shows observed blood pressure reductions of 1.1 mm Hg and 0.9 mm Hg for systolic and diastolic blood pressures, respectively [57].

WO did not differ from WD and WE in mean change in body weight or waist circumference across all phases, a finding that was unexpected and counter to our hypotheses. These results might be due to the robust nature of the web-based intervention used by all three groups. The web-based intervention was comprehensive in that it included a structured weight loss and weight maintenance plan, delivered updated content frequently, provided email prompts when new content was updated, and included self-monitoring for eating, activity, and goal setting across all phases. Such comprehensive web-based features promote greater weight reduction than those with basic informational elements [9, 58]. The frequency of the assessments, found by others to enhance achievement of weight loss or weight maintenance, might have had an unintended effect as intervention boosters particularly for the WO group [16].

The combination of frequency of our assessments with the email prompts when new web content appeared may have been a factor in our relatively high retention rate for all three groups over the 30 months, being 88% or greater at 6 months, with declines to 69% or greater by 30 months. Compared to other studies, our retention was relatively high given the long intervention period. The literature cites a wide range of retention rates for purely web-delivered weight loss, from 6% to 35% [58, 59] over 12 weeks, with others having higher retention of 80% after 12 months of intervention [16].

Issues with long-term sustainability and nonusage attrition are common in web-based interventions and are problems that appear to increase over time [60]. Reasons for disengagement may be motivational, failure to achieve weight loss, or lack of interest in the features or content of the website, which may lead to boredom [60]. Consistent with the literature [61], success at weight loss and weight loss maintenance among our women was correlated with the level of women's engagement by our proxy of intervention dosage, with the pattern of website dosage received being similar across all groups, declining over the three phases. Our method of tracking dosage, being percent of women who logged in at least once when new content was delivered (i.e., weekly, biweekly, or monthly), was similar to that of Cussler and colleagues [15], who defined dosage as the percentage of participants who used the feature at least once per week. Several groups of researchers [7, 59] noted the challenges in comparing adherence data, as there is no standardization for adherence overall, though studies report a positive association between participant adherence and weight change.

Women in the WD group had relatively low engagement in the discussion board feature initially during phase 1 with continued declines over phases 2 and 3. The reasons for this low engagement in the discussion board are unclear as our prior 3-month pilot study comparing WO to WD showed enhanced engagement and weight loss in the WD group. As the women's enrollment in this large trial occurred over a one-year period, it was possible that the women never sensed they were part of a cohort, or they first entered the discussion board at times when the content was not helpful to their given needs or interests, or the technology frustrated them. As noted in a systematic review of online peer-to-peer interactions, there is a lack of robust evidence on peer-to-peer online support, though this does not mean that virtual social communities have no effect, as little is known about factors that might influence outcomes, such as moderator influences and/or individual and group interests [22].

In contrast, women in the WE group appeared more engaged. The percentage of women voluntarily responding to counselor emails was highest during phase 1 (89%) and declined slightly during phase 2 (69%). In phase 3, 16% of women self-initiated an email to counselor when no routine counselor emails were sent. A potential reason for this level of engagement may be attributed to the personalization that is perceived with individualized emails, a finding observed by others [7, 58].

The strengths of this study included its design, comparing web-based only with two other web-based interventions with enhanced features among a population of underserved and understudied women from rural communities. The study included successful recruitment and retention of a large cohort of women from rural communities over 30 months, with nearly half from small rural or isolated rural areas. Analyses of primary outcomes were adequately powered (.80) for a moderate effect size consistent with prior weight loss and maintenance comparisons of interventions. For secondary outcomes, power had not been estimated a priori, but the size of the group difference in proportion needed to be found significant appears to also be meaningful. The uniqueness of this intervention is its inclusion of three phases, providing interventions that targeted guided weight loss, followed by longer intervention periods of 12 months each for guided weight maintenance and self-managed weight maintenance, and tracking women's engagement. The study design and implementation included features established for reporting eHealth interventions [62], which were published after this trial started enrolling participants.

Limitations included the majority of the women being from a relatively high socioeconomic background, commonly reported in other web-based interventions, which may limit generalizability since the findings may not be representative of the population of overweight and obese women from rural communities. Another important limitation is that we cannot know to what extent missing observations were truly missing at random. The nonsignificant difference for weight loss between completers and noncompleters at 18 months provides some assurance that the MAR assumption may be tenable.

Contamination may have occurred between groups due to the women's familiarity and sharing with other women in the rural areas of residence. Though the nurses were blinded to the women's group assignment and to the intervention, the frequent contacts could have influenced the women's behavior for achieving weight loss or influenced retention. The issue of decreased or passive engagement with the intervention over time is common in web-based interventions. Additional analysis of women's engagement with specific website features associated with successful weight loss and weight maintenance may prove insightful.

The implications of this research are that women from rural communities were willing and able to participate in long-term web-based interventions for weight loss and weight maintenance. Future studies might focus on recruiting women more representative of the population, refining the interventions to encourage weight maintenance, and focusing on the cost-effectiveness of implementing web-based only interventions compared to those with supplemental features.

## 6. Conclusions

This study supports that women from rural communities with overweight or obesity were willing to participate in web-based interventions, either web-based only or web-based with supplemental elements, with an estimated 42% (WO), 38% (WD), and 51% (WE) being able to achieve clinically relevant weight loss of ≥5% by six months, with weight regain by half at 30 months, though the WD and WE groups did not result significantly in more improvement compared to the WO group, potentially due to the robust nature of the web-based intervention. Though weight change was modest, the use of web-based interventions may be clinically relevant for reaching rural women on a public health level, as small reductions in weight have been shown to have health benefits. As women from rural communities may have few resources for weight management, web-based weight loss and weight maintenance programs may be potentially important as an alternative venue.

## Supplementary Material

The supplemental material depicts examples of web-based intervention screen views provided to all women during the practice period, phase 1, phase 2, and phase 3. In addition it includes two illustrations of web-screen views specific to the peer-led discussion board group (WD).

## Figures and Tables

**Table 1:** Summary of web-based interventions delivered to web-based only (WO) group, web-based with peer-led discussion (WD) group, and web-based with professional email counseling (WE) group.

Group	Phase 1 (baseline to 6 months) guided weight loss	Phase 2 (6 to 18 Months) weight loss & weight maintenance	Phase 3 (18 to 30 Months) self-directed weight maintenance
All Groups	*Messaging*	*Messaging*	*Messaging*
	Lifestyle weight loss plan^a,b^ New content posted weekly	Lifestyle weight maintenance added Hot topics (i.e., news) posted biweekly Access to phase 1 messages allowed	Preventing weight regain Content Library & success stories Videos monthly, 18 to 24 months Videos bimonthly, 24 to 30 months
	*Targets*	*Targets*	*Targets*
	≥5 to 10% loss initial bodyweight ≥150 weekly minutes of moderate or greater intensity physical activity Ave daily caloric intake ≤ 1,600 Kcal (range 1,200–1,600) Ave daily fat intake (20–35%)	≥5–10% weight loss from baseline or maintenance without regain ≥150 weekly minutes of moderate or greater intensity physical activity increasing to average 60–90 min daily Ave daily caloric intake ≤ 1,600 Kcal Ave daily fat intake (20–35%)	≥5–10% weight loss from baseline or maintenance without regain ≥150 weekly minutes of moderate or greater intensity physical activity increasing to average 60–90 min daily Ave daily caloric intake ≤ 1,600 Kcal Ave daily fat intake (20–35%)
	*Monitoring*	*Monitoring*	*Monitoring*
	Weight logging daily Kcal intake daily, food count book provided^c^ Steps taken daily, pedometer provided Goal setting, weekly	Weight logging weekly Eating and activity logs available biweekly tracking encouraged Goal setting, biweekly	Weight logging weekly encouraged Eating and activity logs available tracking encouraged Goal setting encouraged
	*Feedback*	*Feedback*	*Feedback*
	Feedback from baseline, 3- and 6-month assessments provided online	Feedback from 12- and 18-month assessments provided online	Feedback from 24- and 30-month assessments provided online

WD only	*Discussion board*	*Discussion board*	*Discussion board*
	Peer-led asynchronous discussion with weekly primers posted by peer	Peer-led asynchronous discussion with biweekly primers posted 6 to 12 months and monthly primers posted 12 to 18 months	Access to discussion board with only three primers posted by peer-leader

WE only	*Professional email counseling*	*Professional email counseling*	*Professional email counseling*
	Weekly feedback on women's postings Counselor responds to email questions One reminder sent by counselor to any WE woman who forgot to post	Biweekly feedback on women's postings from 6 to 12 months and once monthly during months 12 to 18 One reminder sent by counselor to any woman who has not posted in scheduled weeks	No counselor feedback on postings is provided Counselor responds to any emails initiated by women

^a^US Department of Health and Human Services and US Department of Agriculture, Dietary Guidelines for Americans 2010.

^b^Office of Disease Prevention and Health Promotion, 2008 Physical Activity Guidelines for Americans.

^c^Netzer CT, *The Complete Book of Food Counts*, 8th Ed., Dell Publishing Company, 2008.

**Table 2:** Study aims, with primary and secondary outcomes, instrumentation, and assessment time points.

Variable	Instrument		Assessment time point						
		Base	3 Mo	6 Mo	9 Mo	12 Mo	18 Mo	24 Mo	30 Mo
*Aim 1: weight loss and weight maintenance*									
Primary outcomes									
Body weight (kg)	Tanita scale (TBF 215)	X	X	X	X	X	X	X	X
Waist circumference (cm)	Tape measure	X	X	X	X	X	X	X	X
Secondary outcomes									
Proportion achieving at least 5% weight loss	Tanita scale (TBF 215)			X			X		X
Proportion achieving at least 10% weight loss	Tanita scale (TBF 215)			X			X		X
*Aim 2: eating and activity*									
Primary outcomes									
Kcal intake daily	Web-version Block Health Habit and History Questionnaire								
		X		X			X		X
Weekly minutes moderate or greater intensity activity	Actigraph Model GT3X Accelerometer	X		X			X		X
Weekly minutes moderate or greater intensity activity	Survey (BRFSS)	X	X	X	X	X	X	X	X
Systolic blood pressure (mmHg)	Automatic Sphygmomanometer ADC e-sphyg™ 2 Model 9002								
		X	X	X	X	X	X	X	X
Diastolic blood pressure (mmHg)	Automatic Sphygmomanometer ADC e-sphyg 2 Model 9002	X	X	X	X	X	X	X	X
Total cholesterol (mL/dL)	Chemical blood analysis	X		X			X		X
HDL (mL/dL)	Chemical blood analysis	X		X			X		X
LDL (mL/dL)	Chemical blood analysis	X		X			X		X
Triglycerides (mL/dL)	Chemical blood analysis	X		X			X		X
Fasting glucose (mL/dL)	Chemical blood analysis	X		X			X		X
Secondary outcomes									
Proportion achieving 20–35% calories from fat	Web-version Block Health Habit and History Questionnaire	X		X			X		X
Proportion achieving ≥ 150 minutes weekly moderate or greater intensity activity	Actigraph Model GT3X Accelerometer	X		X			X		X

**Table 3:** Baseline characteristics of rural women by randomized group.

Variable	Web-based only (WO)		Web-based discussion (WD)		Web-based email (WE)	
	*n* = 101		*n* = 100		*n* = 100	
	*n*	%	*n*	%	*n*	%
Race or ethnicity						
White	99	98%	98	98%	96	96%
Hispanic	1	1%	1	1%	1	1%
Other	1	1%	1	1%	2	1%
No response	0	0%	0	0%	1	1%
Education						
High school or lower	20	20%	12	12%	15	15%
Some college	45	45%	45	45%	41	41%
College grad or above	36	35%	43	43%	44	44%
Employment						
Full time	67	66%	63	63%	77	77%
Part time	18	18%	26	26%	9	9%
Household income						
<$20,000	1	1%	4	4%	3	3%
$20,000 to $39,999	15	15%	15	15%	13	13%
$40,000 to $59,999	25	25%	26	26%	27	27%
$60,000 or higher	52	52%	49	49%	51	51%
Rural residency						
Large rural	56	55%	63	63%	70	70%
Small rural	10	10%	10	10%	4	4%
Isolated	33	33%	27	27%	26	26%
Comorbid conditions						
Diabetes	3	3%	5	5%	6	6%
Respiratory	6	6%	5	5%	7	7%
Arthritis	28	28%	21	21%	31	31%
Other muscular conditions	7	7%	10	10%	15	15%
Cancer	11	11%	9	9%	7	7%
Thyroid	28	28%	20	20%	22	22%
Cardiovascular	3	3%	1	1%	4	4%
General	34	34%	40	40%	31	31%
Smoking cigarettes	1	1%	4	4%	7	7%
General health						
Excellent to very good	38	38%	26	26%	40	40%
Good	54	54%	60	60%	47	47%
Poor to fair	9	9%	13	13%	13	13%
BMI category (kg/m^2^)						
Overweight (25–29.9)	12	12%	7	7%	13	13%
Obese I (30–34.9)	50	50%	49	49%	44	44%
Obese II (35–39.9)	24	24%	30	30%	31	31%
Obese III (≥40 to 45)	15	15%	14	14%	12	12%

**Table 4:** Observed means of primary outcome measures of intervention groups.

Variable	Web-based only (WO)		Web-based discussion (WD)		Web-based email (WE)	
	*n* = 101		*n* = 100		*n* = 100	
	*n*	Mean (SD)	*n*	Mean (SD)	*n*	Mean (SD)
Body weight (kg)						
Baseline	101	93.6 (13.7)	100	94.5 (12.9)	100	93.3 (12.6)
6 months	87	88.0 (14.6)	88	89.4 (13.9)	92	87.2 (13.5)
18 months	81	88.9 (14.8)	72	89.6 (13.6)	77	88.3 (15.5)
30 months	76	89.4 (14.0)	67	90.4 (13.3)	73	89.5 (16.7)
Waist circumference (cm)						
Baseline	101	109.4 (11.6)	100	108.8 (10.9)	100	108.5 (10.0)
6 months	87	102.8 (12.1)	88	103.3 (11.6)	91	102.4 (11.2)
18 months	81	104.4 (12.0)	71	104.1 (11.3)	75	103.8 (13.4)
30 months	76	103.8 (12.0)	66	103.8 (10.6)	73	104.3 (13.6)
Kcal intake daily						
Baseline	101	1809 (729)	100	1930 (675)	100	1789 (556)
6 months	85	1516 (535)	88	1514 (513)	91	1415 (447)
18 months	79	1532 (587)	70	1527 (610)	73	1371 (534)
30 months	75	1473 (499)	65	1384 (434)	72	1375 (511)
Weekly minutes ≥ moderate intensity activity (Actigraph)						
Baseline	100	274.6 (149.6)	99	252.0 (125.0)	100	227.2 (137.3)
6 months	86	299.1 (165.3)	84	273.0 (154.5)	87	239.2 (126.0)
18 months	78	260.3 (133.5)	71	251.8 (137.7)	72	219.6 (113.3)
30 months	72	267.3 (133.1)	65	248.4 (150.6)	68	231.5 (131.0)
Weekly minutes ≥ moderate intensity activity (BRFSS)						
Baseline	101	127.8 (197.9)	100	104.4 (135.6)	100	109.7 (139.6)
6 months	87	183.9 (232.1)	88	133.4 (172.5)	91	171.2 (274.7)
18 months	81	123.6 (146.5)	71	125.1 (188.4)	75	125.9 (186.2)
30 months	76	119.4 (160.8)	66	108.0 (171.2)	73	100.4 (131.1
Systolic blood pressure (mmHg)						
Baseline	101	123.4 (12.6)	100	121.4 (10.9)	100	124.4 (12.3)
6 months	87	120.5 (16.3)	88	120.1 (12.5)	91	120.6 (13.0)
18 months	81	122.0 (17.0)	71	120.1 (11.6)	75	121.3 (13.2)
30 months	76	122.0 (13.0)	66	120.7 (13.3)	73	122.0 (14.7)
Diastolic blood pressure (mmHg)						
Baseline	101	76.9 (7.9)	100	76.5 (7.8)	100	77.0 (8.2)
6 months	87	75.8 (8.7)	88	75.6 (9.0)	91	75.6 (8.4)
18 months	81	76.0 (9.1)	71	75.6 (7.8)	75	74.8 (9.6)
30 months	76	75.7 (7.7)	66	74.8 (8.4)	73	74.7 (8.8)
Total cholesterol (mL/dL)						
Baseline	100	196.8 (36.8)	99	200.4 (37.4)	100	200.4 (43.7)
6 months	86	199.6 (41.8)	88	204.7 (36.9)	92	192.0 (45.1)
18 months	81	200.0 (32.7)	72	211.7 (35.0)	74	198.7 (39.9)
30 months	73	201.7 (33.5)	66	210.5 (35.8)	71	196.6 (40.8)
HDL (mL/dL)						
Baseline	101	48.5 (10.6)	99	50.5 (13.2)	100	49.8 (14.6)
6 months	86	53.0 (11.7)	88	53.7 (11.3)	92	50.0 (13.0)
18 months	81	56.1 (12.5)	72	56.6 (11.3)	74	54.6 (11.0)
30 months	73	56.1 (10.8)	66	58.5 (14.8)	71	54.1 (12.0)
LDL (mL/dL)						
Baseline	101	121.7 (32.4)	98	124.8 (32.5)	100	122.2 (35.3)
6 months	86	122.8 (34.4)	88	127.7 (32.4)	92	116.9 (37.0)
18 months	80	117.5 (30.0)	71	129.2 (29.6)	74	119.5 (36.5)
30 months	73	119.3 (29.9)	64	126.3 (29.7)	71	116.0 (35.1)
Triglycerides (mL/dL)						
Baseline	101	134.7 (68.6)	99	129.6 (87.5)	100	141.6 (69.2)
6 months	86	118.8 (60.3)	88	116.7 (53.9)	92	125.6 (63.0)
18 months	81	136.0 (76.4)	72	129.4 (75.1)	74	122.9 (55.4)
30 months	73	131.8 (63.9)	66	128.9 (72.7)	71	132.7 (60.2)
Fasting glucose (mL/dL)						
Baseline	101	101.7 (15.5)	99	102.0 (22.2)	100	99.4 (14.2)
6 months	86	87.7 (14.5)	88	100.9 (21.4)	92	99.0 (15.8)
18 months	81	97.7 (11.5)	72	96.3 (12.2)	74	97.7 (14.2)
30 months	73	99.6 (12.2)	66	100.5 (19.9)	70	97.7 (16.5)

Note: 6, 18, and 30 months corresponded to the end of phases 1, 2, and 3, respectively.

**Table 5:** Linear mixed model^a^ tests of pairwise group comparisons of estimated difference in change by phase of intervention.

Outcome	Phase 1		Phase 2		Phase 3	
	Baseline to 6 months		6 months to 18 months		18 months to 30 months	
Pairwise comparison	*p* ^b^	Estimated mean difference (95% CI)	*p* ^b^	Estimated mean difference (95% CI)	*p* ^b^	Estimated mean difference (95% CI)
Body weight (kg)^c^						
WO versus WD	0.138	0.9 (−0.8 to 2.7)	0.360	0.2 (−1.1 to 1.6)	0.268	−0.4 (−1.8 to 0.9)
WO versus WE	0.188	−0.8 (−2.5 to 0.9)	0.411	−0.2 (−1.5 to 1.2)	0.444	−0.1 (−1.4 to 1.2)
WD versus WE	0.047	−1.7 (−3.4 to 0.0)	0.563	−0.4 (−1.7 to 1.0)	0.632	0.3 (−1.0 to 1.7)
Waist circumference (cm)^c^						
WO versus WD	0.070	1.6 (−0.5 to 3.7)	0.497	−0.01 (−1.5 to 1.4)	0.479	−0.04 (−1.5 to 1.4)
WO versus WE	0.461	0.1 (−2.0 to 2.2)	0.441	−0.1 (−1.5 to 1.3)	0.463	−0.1 (1.5 to 1.3)
WD versus WE	0.164	−1.5 (−3.5 to 0.6)	0.891	−0.1 (−1.6 to 1.4)	0.970	−0.03 (−1.5 to 1.4)
Kcal intake daily						
WO versus WD	0.105	−96.9 (−248.7 to 55.0)	0.473	−4.7 (−138.8 to 129.3)	0.134	−67.3 (−186.5 to 52.0)
WO versus WE	0.139	−83.5 (−234.6 to 67.7)	0.289	−37.6 (−170.5 to 95.2)	0.183	53.6 (−62.8 to 170.0)
WD versus WE	0.861	13.4 (−137.2 to 164.1)	0.633	−32.9 (−168.4 to 102.6)	0.050	120.9 (0.0 to 241.8)
Weekly minutes ≥ moderate intensity activity (Actigraph)						
WO versus WD	0.497	−0.2 (−38.3 to 37.9)	0.271	10.9 (−24.2 to 46.0)	0.373	−6.0 (−42.2 to 30.2)
WO versus WE	0.332	−8.3 (−46.0 to 29.3)	0.270	10.9 (−24.1 to 45.9)	0.240	12.9 (−23.0 to 48.7)
WD versus WE	0.672	−8.2 (−46.1 to 29.8)	0.999	0.02 (−35.6 to 35.7)	0.313	18.9 (−17.9 to 55.6)
Weekly minutes ≥ moderate intensity activity (BRFSS)^c^						
WO versus WD	0.207	−30.6 (−104.2 to 43.0)	0.055	51.6 (−11.7 to 114.8)	0.344	−10.1 (−59.8 to 39.6)
WO versus WE	0.427	6.8 (−66.4 to 80.0)	0.336	13.5 (−49.1 to 76.1)	0.288	−13.8 (−62.5 to 34.8)
WD versus WE	0.314	37.4 (−35.6 to 110.5)	0.238	−38.1 (−101.4 to 25.2)	0.885	−3.7 (−54.2 to 46.7)
Systolic blood pressure (mmHg)^c^						
WO versus WD	0.209	1.3 (−1.9 to 4.6)	0.318	−0.9 (−4.8 to 2.9)	0.419	0.4 (−3.3 to 4.0)
WO versus WE	0.137	−1.8 (−5.1 to 1.4)	0.395	−0.5 (−4.3 to 3.3)	0.310	0.9 (−2.7 to 4.5)
WD versus WE	0.054	−3.2 (−6.4 to 0.1)	0.833	0.4 (−3.4 to 4.3)	0.782	0.5 (−3.2 to 4.2)
Diastolic blood pressure (mmHg)^c^						
WO versus WD	0.317	0.5 (−1.4 to 2.3)	0.431	−0.2 (−2.4 to 2.0)	0.468	−0.1 (−2.2 to 2.1)
WO versus WE	0.216	−0.7 (−2.6 to 1.1)	0.215	−0.9 (−3.1 to 1.3)	0.342	0.4 (−1.7 to 2.5)
WD versus WE	0.205	1.2 (−3.0 to 0.7)	0.548	−0.7 (−2.9 to 1.6)	0.639	0.5 (−1.7 to 2.7)
Total cholesterol (mL/dL)						
WO versus WD	0.473	0.4 (−10.2 to 11.0)	0.094	7.2 (−3.5 to 17.9)	0.154	−4.3 (−12.7 to 4.0)
WO versus WE	0.026	−10.4 (−20.8 to 0.1)	0.076	7.7 (−2.8 to 18.3)	0.116	−5.0 (−13.2 to 3.2)
WD versus WE	0.043	−10.8 (−21.2 to −0.3)	0.918	0.6 (−10.2 to 11.3)	0.873	−0.7 (−9.2 to 7.8)
HDL (mL/dL)						
WO versus WD	0.060	−2.2 (−5.0 to 0.6)	0.365	0.4 (−2.1 to 3.0)	0.147	1.4 (−1.2 to 4.1)
WO versus WE	0.002^d^	−4.0 (−6.8 to −1.3)	0.062	2.0 (−0.5 to 4.4)	0.273	−0.8 (−3.4 to 1.8)
WD versus WE	0.196	−1.8 (−4.6 to 0.9)	0.241	1.5 (−1.0 to 4.0)	0.105	−2.2 (−4.9 to 0.5)
LDL (mL/dL)						
WO versus WD	0.350	1.6 (−6.7 to 10.0)	0.081	6.1 (−2.5 to 14.8)	0.064	−5.6 (−12.7 to 1.6)
WO versus WE	0.079	−5.9 (−14.1 to 2.3)	0.023	8.7 (0.2 to 17.2)	0.029	−6.8 (−13.8 to 0.2)
WD versus WE	0.072	−7.5 (−15.8 to 0.7)	0.561	2.6 (−6.1 to 11.3)	0.732	−1.3 (−8.5 to 6.0)
Triglycerides (mL/dL)						
WO versus WD	0.370	3.1 (−15.0 to 21.1)	0.429	−1.4 (−16.6 to 13.8)	0.337	3.2 (−11.9 to 18.4)
WO versus WE	0.477	0.5 (−17.4 to 18.4)	0.028	−14.7 (−29.8 to 0.3)	0.023	15.3 (0.4 to 30.3)
WD versus WE	0.782	−2.5 (−20.4 to 15.4)	0.089	−13.3 (−28.7 to 2.0)	0.123	12.1 (−3.3 to 27.5)
Fasting glucose (mL/dL)						
WO versus WD	0.081	2.7 (−1.1 to 6.5)	0.141	−2.0 (−5.7 to 1.7)	0.360	0.6 (−2.9 to 4.2)
WO versus WE	0.024	3.8 (0.1 to 7.6)	0.195	−1.6 (−5.2 to 2.0)	0.046	−3.0 (−6.4 to 0.5)
WD versus WE	0.565	1.1 (−2.7 to 4.9)	0.827	0.4 (−3.3 to 4.1)	0.047	−3.6 (−7.2 to 0.0)

^a^Direct maximum likelihood methods were used to analyze a mixed-effects repeated-measures model (random intercept, unstructured variance/covariance matrix), with tests of pairwise contrasts of group differences in the change in outcome from beginning to end of each phase.

^b^Comparisons of web-based with discussion board (WD) and web-based with email counseling (WE) with web-based only (WO) were one-tailed and WD with WE were two-tailed, each tested at *α* = .017.

^c^Analyses also included data collected at 3, 12, and 24 months, but comparisons focused on group differences in change from the beginning to the end of each phase.

^d^Significant effect was not in hypothesized direction.

**Table 6:** Estimated proportion^a^ meeting criteria in each intervention group, tests of pairwise group comparisons^b^, and odds ratios at 6 months, 18 months, and 30 months.

Outcome	Estimated proportions			WO versus WD		WO versus WE		WD versus WE	
	WO	WD	WE	*p* ^c^	OR (95% CI)	*p* ^c^	OR (95% CI)	*p* ^c^	OR (95% CI)
Weight loss ≥ 5% of baseline									
6 months	.42	.38	.51	0.290	1.2 (0.7 to 2.2)	0.117	0.7 (0.4 to 1.3)	0.078	1.7 (0.9 to 3.0)
18 months	.31	.31	.42	0.476	1.0 (0.5 to 2.1)	0.066	0.6 (0.3 to 1.2)	0.158	1.7 (0.8 to 3.4)
30 months	.32	.31	.40	0.442	1.1 (0.5 to 2.2)	0.135	0.7 (0.4 to 1.3)	0.239	1.5 (0.8 to 3.0)
Weight loss ≥ 10% of baseline									
6 months	.24	.19	.29	0.297	1.3 (0.6 to 2.7)	0.206	0.8 (0.4 to 1.5)	0.120	1.7 (0.9 to 3.4)
18 months	.17	.17	.18	0.433	1.1 (0.5 to 2.5)	0.311	0.8 (0.4 to 1.8)	0.528	1.3 (0.6 to 3.0)
30 months	.29	.20	.20	0.326	0.8 (0.3 to 2.0)	0.185	0.7 (0.3 to 1.5)	0.700	1.2 (0.5 to 2.7)
20–35% calories from fat									
6 months	.53	.38	.56	0.023	1.8 (1.0 to 3.4)	0.341	0.9 (0.5 to 1.6)	0.017	2.1 (1.1 to 3.8)
18 months	.48	.32	.45	0.019	1.9 (1.0 to 3.6)	0.346	1.1 (0.6 to 2.2)	0.109	1.7 (0.9 to 3.3)
30 months	.38	.29	.46	0.096	1.5 (0.8 to 2.9)	0.151	0.7 (0.4 to 1.3)	0.021	2.1 (1.1 to 4.0)
≥150 minutes weekly moderate or greater intensity activity (Actigraph)									
6 months	.78	.81	.75	0.279	0.8 (0.4 to 1.7)	0.361	1.1 (0.6 to 2.3)	0.352	0.7 (0.3 to 1.5)
18 months	.79	.71	.71	0.138	1.5 (0.7 to 3.1)	0.131	1.5 (0.7 to 3.3)	0.940	1.0 (0.5 to 1.9)
30 months	.74	.68	.64	0.236	1.3 (0.6 to 2.7)	0.093	1.6 (0.8 to 3.1)	0.591	0.8 (0.4 to 1.7)
≥150 minutes weekly moderate or greater intensity activity (BRFSS)									
6 months	.44	.36	.42	0.159	1.4 (0.7 to 2.7)	0.403	1.1 (0.6 to 2.0)	0.397	1.3 (0.7 to 2.4)
18 months	.34	.32	.36	0.443	1.1 (0.5 to 2.2)	0.361	0.9 (0.5 to 1.7)	0.623	1.2 (0.6 to 2.4)
30 months	.31	.32	.27	0.444	1.0 (0.5 to 1.9)	0.286	1.2 (0.6 to 2.4)	0.489	0.8 (0.4 to 1.6)

*Notes*. WO is web-based only group, WD is web-based with peer-led discussion group, and WE is web-based with professional email counseling.

^a^Proportions estimated from the 20 imputed datasets.

^b^Generalized estimating equations (GEEs) were used with multiply imputed dataset to fit a model to all three times, incorporating tests of pairwise comparisons of groups on the proportion meeting criteria at end of each phase.

^c^WO versus WD and WO versus WE are one-tailed tests to match hypotheses, and WD versus WE is two-tailed, each tested at *α* = .017.

**Table 7:** Dosage of web-delivered content as assessed by women's logins at time points when new intervention content was posted by intervention phase.

Dosage^a^	Web-based only (WO)		Web-based discussion (WD)		Web-based email (WE)	
Dosage operationally defined when women logged into website during time points when new content was available, regardless of number of logins	*n*	%	*n*	%	*n*	%
*Web-delivered content to all groups *						
Phase 1^b^, 26 segments of new content posted over 26 weeks						
100% dosage (26 segments)	16	18.4%	11	12.5%	12	13.0%
75–99% dosage (20–25 segments)	21	24.1%	32	36.4%	30	32.6%
50–74% dosage (13–19 segments)	23	26.4%	15	17.0%	22	23.9%
25–46% dosage (7–12 segments)	14	16.1%	16	18.2%	12	13.0%
1–25% dosage (1–6 segments)	13	14.9%	13	14.8%	15	16.3%
0% no dosage (0 segments)	0	0.0%	1	1.1%	1	1.1%
Total number of phase 1 completers	87		88		92	
Phase 2^c^, 26 segments of new content posted over 1 year						
100% dosage (26 segments)	3	3.7%	0	0.0%	2	2.6%
75–99% dosage (20–25 segments)	13	16.0%	9	12.5%	15	19.5%
50–74% dosage (13–19 segments)	6	7.4%	8	11.1%	9	11.7%
25–46% dosage (7–12 segments)	14	17.3%	10	13.9%	8	10.4%
1–25% dosage (1–6 segments)	28	34.6%	28	38.9%	29	37.7%
0% no dosage (0 segments)	17	21.0%	17	23.6%	14	18.2%
Total number of phase 2 completers	81		72		77	
Phase 3^d^, 9 segments of new content posted over 1 year						
>55.5%–100% dosage (6–9 segments)	16	21.1%	17	25.3%	15	20.5%
1–55.5% dosage (1–5 segments)	54	71.0%	40	59.7%	54	73.97%
0% no dosage (0 segments)	6	7.9%	10	14.9%	5	6.8%
Total number of phase 3 completers	76		67		73	
*Web-based discussion board primer content (WD)* ^e^						
Phase 1, 26 new discussion board primers posted over 26 weeks						
100% dosage (26 primers)			8	9.1%		
75–99% dosage (20–25 primers)			19	21.6%		
50–74% dosage (13–19 primers)			13	14.8%		
25–46% dosage (7–12 primers)			20	22.7%		
1–25% dosage (1–6 primers)			23	26.1%		
0% no dosage (0 primers)			5	5.7%		
Phase 2, 18 new discussion board primers posted over 1 year						
50–100% dosage (9–18 primers)			16	22.2%		
1–49% dosage (1–8 primers)			34	47.2%		
0% no dosage (0 primers)			22	30.5%		
Phase 3, views and posts						
One or more views			28	41.8%		
Did not view board in phase 3			39	58.2%		
One or more posts			10	14.9%		
Did not post on board in phase 3			57	79.2%		

^a^Dosage was operationally defined as met when women logged on to website during time points when new content was made available, regardless of the number of logins during or between that time point. Note that women received an email prompt when new content was made available.

^b^Phase 1 (baseline to 6 months) offered new content weekly for a total of 26 segments of new content.

^c^Phase 2 (6 to 18 months) offered biweekly new content, named “hot topics” as reflected of current news stories, for a total of 26 segments of new content.

^d^Phase 3 (18 to 30 months) offered new content in form of videos, success stories, and cartoons monthly for months 18 to 24 and bimonthly for months 24 to 30 for a total of 9 segments of new content.

^e^The discussion board peer-leader was responsible for posting new discussion topics called primers on the following schedule: phase 1 primers posted weekly for 26 total; phase 2 primers posted biweekly months 6 to 12 and monthly during months 12 to 18 for 18 total; and, phases 1 and 2 dosage reflected logins regardless of whether women posted or viewed posts. At phase 3, women received 3 initial primers yet had access to discussion board throughout the phase. Only the number of views and the number of posts are reported for WD during phase 3.
