# Supplementary material for: Web-Based Interventions Alone or Supplemented with Peer-Led Support or Professional Email Counseling for Weight Loss and Weight Maintenance in Women from Rural Communities: Results of a Clinical Trial
Source: J Obes. 2017 Apr 5;2017:1602627. doi: 10.1155/2017/1602627 (PMC5396444; doi:10.1155/2017/1602627)

Journal of Obesity

Manuscript 1602627

Additional file provided with this submission:

Additional file 1. Trial Website Screen capture.pdf

Supplemental Figure 1. Trial Website Screen Capture: Web-site screen views for Women Weigh-in for Wellness clinical trial

Practice Period – Prior to Randomization to Groups

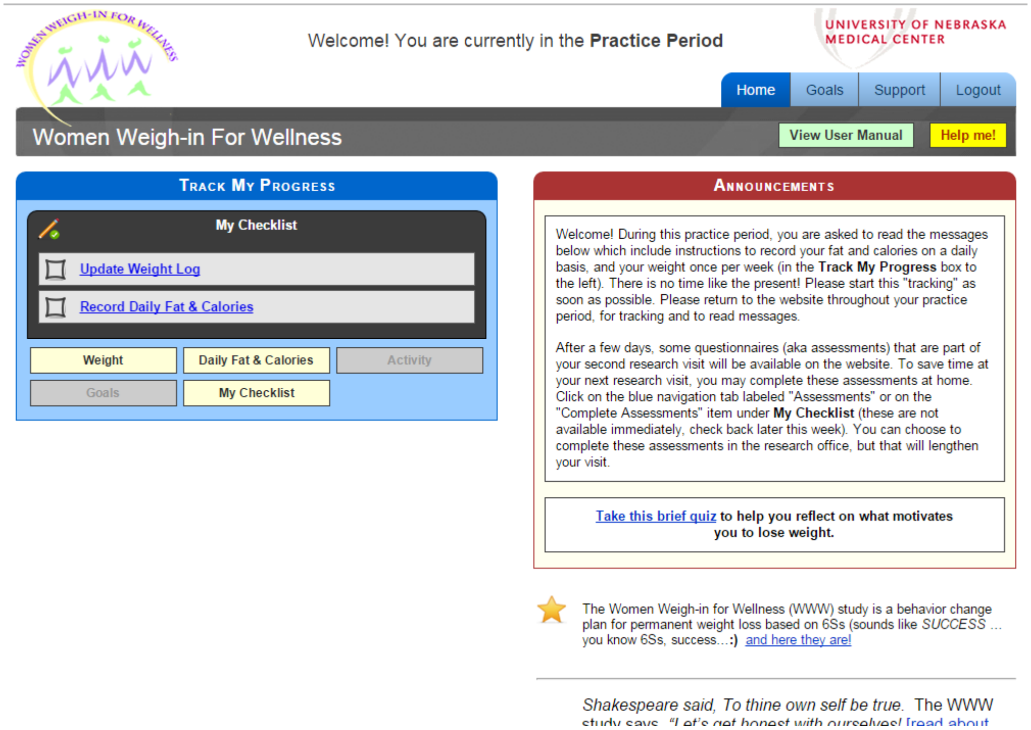

Phase 1 – Example of web-based screen view available to all groups

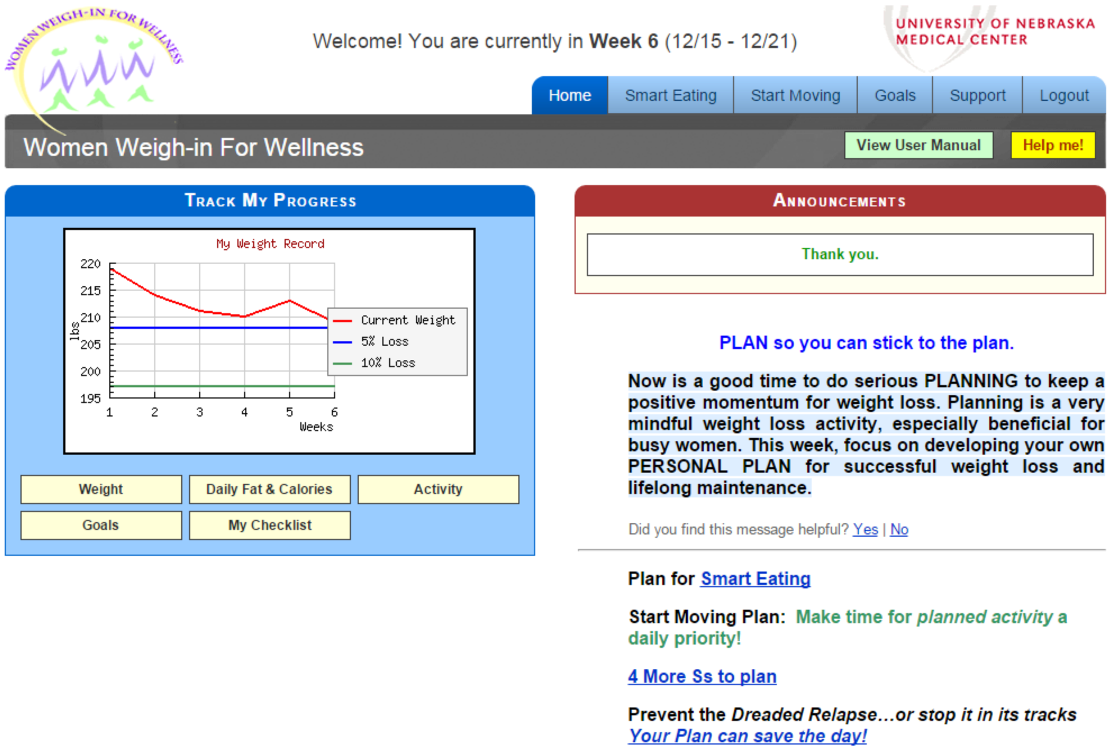

Supplemental Figure 1. Trial Website Screen Capture: Web-site screen views for Women Weigh-in for Wellness clinical trial

Phase 1 – View for WD group illustrating Discuss Tab

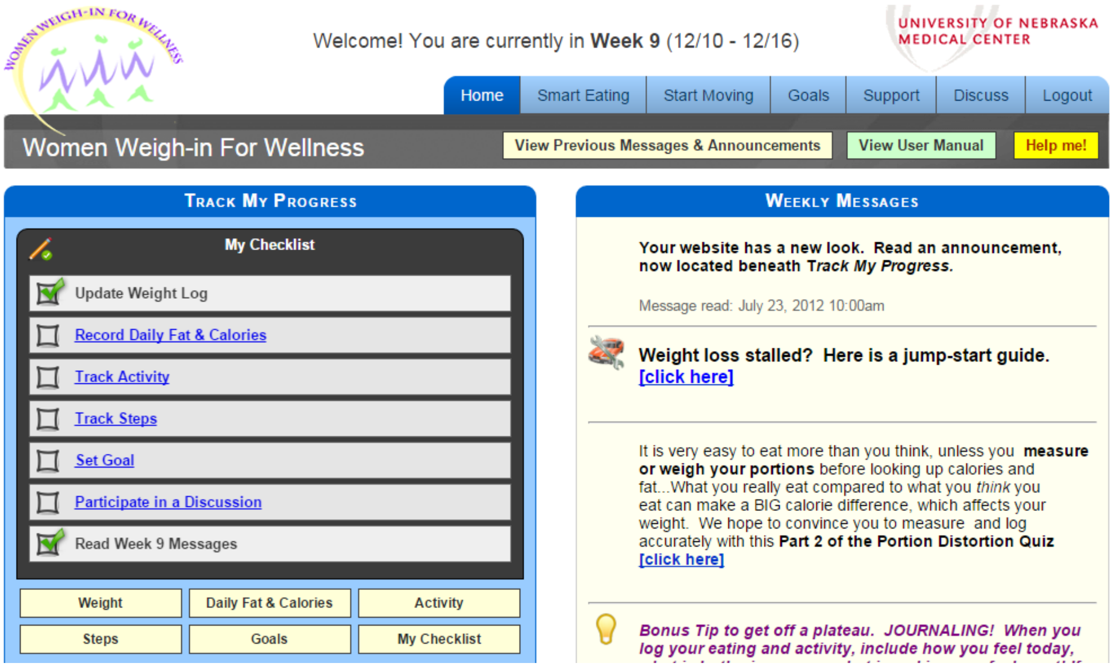

Phase 1 – View of Discussion Board for WD group

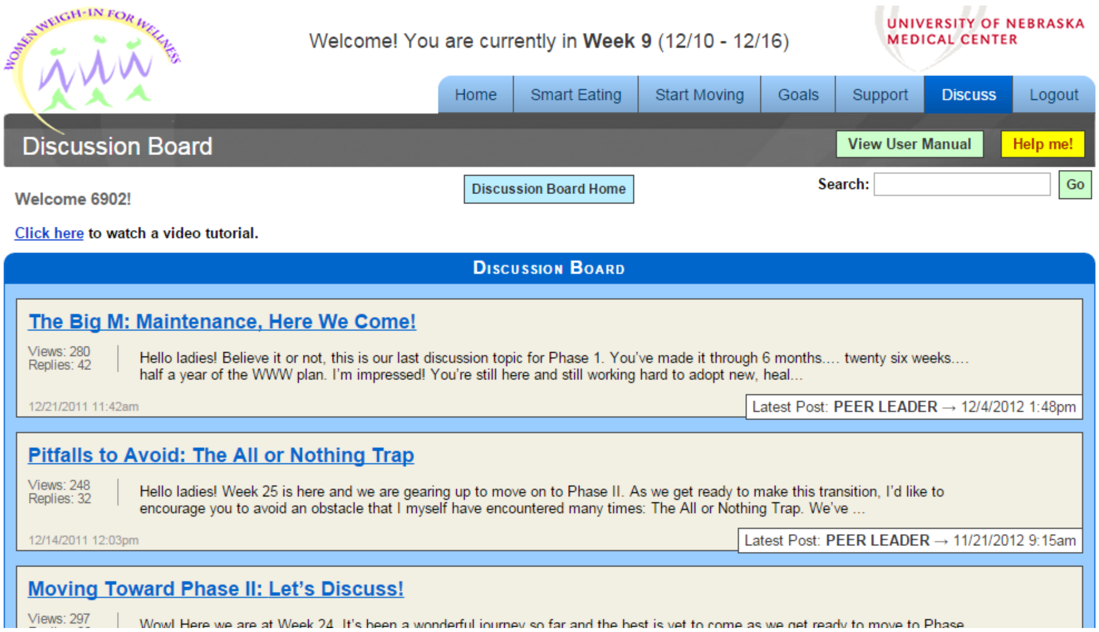

Supplemental Figure 1. Trial Website Screen Capture: Web-site screen views for Women Weigh-in for Wellness clinical trial

Phase 2 - Hot Topic Example available to all groups

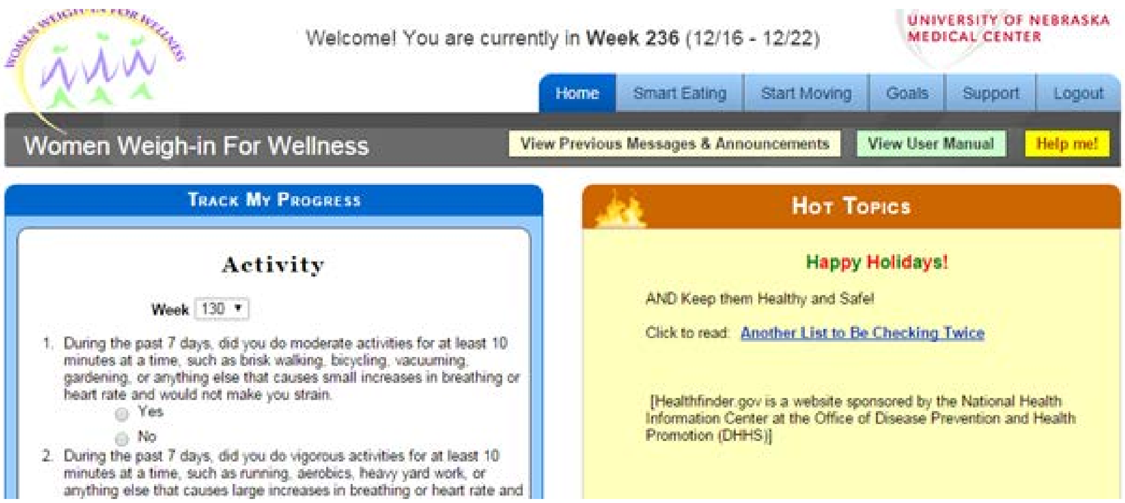

Phase 3 – Home page available to all groups

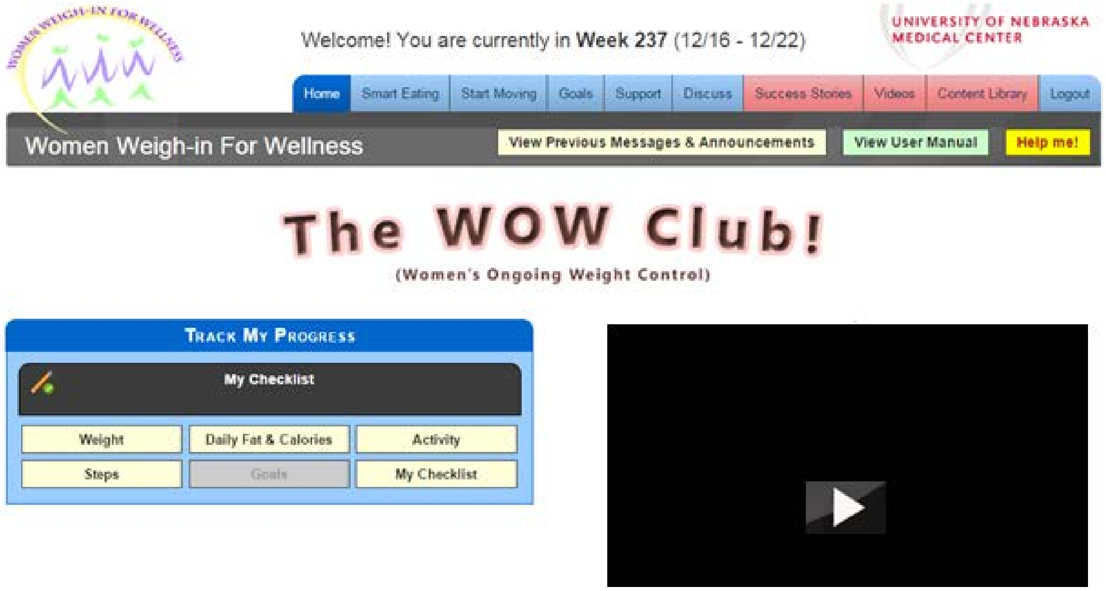

Supplement: Supplementary file 1 — The supplemental material depicts examples of web-based intervention screen views provided to all women during the practice period, phase 1, phase 2, and phase 3. In addition it includes two illustrations of web-screen views specific to the peer-led discussion board group (WD). [file 1602627.f1.pdf]
